# Characteristics of Bronchiectasis Associated with Chronic Obstructive Pulmonary Disease

**DOI:** 10.2174/18743064-v16-e2203311

**Published:** 2022-05-31

**Authors:** Khaled Deghdegh, Nassim Boukadoum, Besma Terra, Kamel Amoura, Rachid Benali

**Affiliations:** 1 Department of Pulmonary Diseases, Faculty of Medicine, Badji Mokhtar University, Annaba, Algeria; 2 Department of Radiology, Faculty of Medicine, Badji Mokhtar University, Annaba, Algeria; 3 Central Biology Laboratory, CAC, Faculty of Medicine, Badji Mokhtar University, Annaba, Algeria

**Keywords:** COPD, Bronchiectasis, Characteristics, Association, Comorbidity, Sputum

## Abstract

**Introduction:**

An association between chronic Obstructive Pulmonary Disease (COPD) and bronchiectasis has been observed. However, the incidence of this association is variable. The current use of high-resolution Chest Thoracic (CT) scans in patients with COPD has contributed to its demonstration. It is comorbidity or even an overlap syndrome. The aim of this study is to determine the characteristics of bronchiectasis in patients with COPD at the Annaba University Hospital in Algeria.

**Subjects and Methods:**

We prospectively included patients hospitalized at the Annaba University Hospital in Algeria between 1^st^ January, 2013 to 31^st^ December, 2015. All patients were hospitalized for an exacerbation of COPD. One hundred and twelve patients (108 men and 04 women) were enrolled in this study. These patients were classified into two groups: G1 (COPD without bronchiectasis) and G2 (COPD with bronchiectasis). The parameters considered for both the groups were the length of hospitalization, comorbidities as per the Charlson index, number of exacerbations in the previous year, quality of life assessed by the Saint Georges questionnaire, a spirométrie, and C. reactive protein (CRP). The diagnosis of exacerbation, bronchiectasis, and COPD was made. The data collected were statistically analyzed using SPSS/version 16. Parametric data were expressed as mean ± SD, and non-parametric data were expressed as the number and percentage of the total. In all tests, a p-value <0.05 was considered significant. Multivariate logistic regression analysis was performed for statistically significant variables.

**Results and Discussion:**

One hundred and twelve patients (108 men and 04 women) were included in the study. The demographic characteristics of the patients were: age (69.49 years ± 8.15), smoking (53.21 ± 48 p/y), and FEV_1_(42.55 ± 18.90 l/s). Of the 112 cases included, 21 had COPD associated with bronchiectasis (18.75%). This bronchiectasis was of cylindrical type in the majority of patients, *i.e*., 17 (80.95%), saccular in 03 cases (14.28%), and mixed in only one case (04.77%). Lesions were bilateral in 16 patients (76.19%) and unilateral in 05 patients (23.81%). FEV_1_ in the COPD group associated with bronchiectasis was more severe (48.7 ± 6 l/s vs. 40.2 ± 3 l/s) (OR=4.3187; 95% C.I **=**2.6301 - 6.8740; p <0.017). Furthermore, we noted that the length of hospitalization, the exacerbations during the past year, the CRP rate, the total score of the Saint Georges questionnaire, and the sputum purulence were statistically significant variables with an Odds Ratio significant in the COPD bronchiectasis association

**Conclusion:**

The diagnosis of bronchiectasis should be more efficient in patients with COPD with a severe respiratory deficit, purulent sputum, accelerated CRP, and deterioration in the quality of life.

## 1 INTRODUCTION

Chronic obstructive pulmonary disease (COPD), by its frequency, severity, and cost, constitutes a public health problem [1]. Patients with different COPD phenotypes may experience a varying natural history of their disease, including the association of COPD with bronchiectasis. Bronchiectasis (from the Greek word “bronkhos,” meaning windpipe or latterly bronchial tubes, and “ektasis,” means stretching out, extension, or dilation [2]. Bronchiectasis is defined as an abnormal, permanent, and irreversible increase in the size of the bronchi due to chronic inflammation, secondary to poor mucociliary clearance, or recurrent or chronic infections [3]. Bronchiectasis is considered by the GOLD (guidelines obstructive lung diseases) 2014 as comorbidity. Due to the common use of high-resolution computed tomography (HRCT) in COPD, bronchiectasis is now more detectable. The two pathologies are characterized by common symptoms, including cough, expectoration, and the possibility of exacerbation due to a recent or persistent infection. They constitute an overlap syndrome [4]. The existence of bronchiectasis in COPD increases the severity of symptoms and the number of exacerbations [5], severe disease, worse outcomes, and more isolation of potentially pathogenic microorganisms [6]. The prevalence of this coexistence varies from 4 to 72% [7] and is observed mainly in the advanced forms of COPD [8]. However, the causes and mechanisms of bronchiectasis in COPD are still not clear.

This study aimed to determine the frequency, characteristics, and impact of bronchiectasis, and in severe cases, meantime of hospital stay, and quality of life in patients with COPD. This study was conducted in the department of pneumology at the university hospital of Annaba City in East Algeria.

## 2 SUBJECTS AND METHODS

### 2.1 Study Subjects

We prospectively included all patients hospitalized for an exacerbation of COPD at the Annaba university hospital in Algeria between 1^st^ January, 2013 to 31^st^ December, 2015. We excluded patients who had bronchiectasis before COPD, asthma, pulmonary fibrosis, and those who had previously been diagnosed with pulmonary tuberculosis. Tuberculosis is the largest cause of bronchiectasis in Algeria; the tuberculosis origin of bronchiectasis was ruled out by questioning, which did not find any history of taking anti-tuberculosis drugs, and by the absence of names of patients included in the district tuberculosis mandatory notification register. One hundred and twelve patients (108 men and 04 women) were enrolled. These patients were classified into 02 groups: G1 (COPD without bronchiectasis) and G2 (COPD with bronchiectasis). At discharge, the length of hospitalization of each patient, as well as the number of COPD exacerbations per year, was noted. One month after their discharge from the hospital and during the COPD check-up, spirometry was performed for each patient to assess the severity of the disease. According to the COPD diagnosis and recommendations of GOLD 2013, we used an easy-one spirometer with calibration. The evaluation of the quality of life was made by the specific questionnaire of Saint George (SGQR) in a validated Arabic version. The questionnaire consisted of two parts (The 2^nd^ one had 07 sections) and three areas (impact, symptoms, and disability). We calculated the score of each patient on 100 points, with an overall score that was calculated by the software. BMI in m^2^ / kg and smoking in terms of the number of packs per year were calculated for each patient. The Anthonisen index was assessed and classified into 03 levels at the time of hospitalization for exacerbation of COPD and compared in both groups. Dyspnea was assessed on exertion according to the mMRC scale (modified Medical Research Council). The average length of hospitalization was calculated (the number of days of hospitalization for exacerbation in each group). C-reactive protein CRP was observed in all patients. All these parameters were compared in the two groups

### 2.2 High-resolution Chest Thoracic Scan

The examination was performed in the supine position during apnea with slices <1mm thick to explore the entire pulmonary field. The diagnosis of bronchiectasis is based on the increase in the diameter of the bronchus relative to the satellite vessel.

### 2.3 Diagnosis Criteria of COPD, Exacerbation, and Bronchiectasis

#### 2.3.1 The Diagnosis of COPD

It is based on the 2013 GOLD criteria: the presence of an obstructive ventilatory disorder (TVO) that is not completely reversible on spirométrie and is defined by a maximum expiratory volume at the first second (FEV1) / forced vital capacity (FVC) ratio) <70% post

Bronchodilatation. Severity is classified according to the FEV1.

#### 2.3.2 Exacerbation of COPD

It is worsening of respiratory symptoms beyond daily variations requiring modification of treatment.

#### 2.3.3 Bronchiectasis

It is defined according to the criteria of Naidich [9]. The 03 morphological aspects of bronchiectasis include cylindrical, tubular, and saccular. It also has a unilateral or bilateral character.

### 2.4 Statistical Analysis

The data collected were statistically analyzed using SPSS/version 16. Parametric data were expressed as mean ± SD, and nonparametric data were expressed as the number and percentage of the total. A student t-test for quantitative independent variables was performed for the analysis of the difference between the two groups. A Chi-square test of significance was used to compare the proportion between the two categorical variables. In all tests, a p-value of <0.05 was considered significant. Multivariate logistic regression analysis was performed for statistically significant variables associated with the presence of bronchial dilations associated with COPD, making it possible to identify the factors independently linked to the presence of bronchiectasis by calculating the Odds Ratio (OR).

### 2.5 Ethical Aspect

Written consent from the institution's ethics committee was obtained and was carried out in compliance with all ethical measures during the various stages of this work, including respect for confidentiality, physical integrity, and patient consent.

## 3 RESULTS

One hundred and twelve patients (108 men and 04 women) were included in the study. The demographic characteristics of the participants are shown in Table **1** including the average age of 69.49 years ± 8.15, smoking of 53.21 ± 48 P / A on average, and an FEV1 of 42.55 ± 18.90 l / s. COPD was recorded at 22.5% as moderate, 43.6% as severe, and 33.9% as very severe. The average six-minute walk test was 273,79 ± 112, 79 m. BODE index was 5, 54± 2, 63. Of the 112 cases included, 21 had COPD associated with bronchiectasis (18.75%), as shown in Fig. (**1**).

Bronchiectasis occurred in the lower lobes in 15 cases (71.42%) and the upper lobes in 06 cases (28.58%). It was the cylindrical type of bronchiectasis in the majority of patients, *i.e*., 17 (80.95%), saccular in 03 cases (14.28%), and mixed in only one case (04.77%). Lesions were bilateral in 16 patients (76.19%) and unilateral in 05 (23.81%) cases, as shown in Table **2**.

Table **3** shows the analysis of the characteristics of COPD in the two groups (G1: 91 cases of COPD without bron-chiectasis and G2: 21 cases associated with bronchiectasis).

The mean age was 70.69 ± 3.37 y in G1 and 68.28 ± 9.14 y in G2. Regarding sex, only 04 female cases were found, all in group 1. The average smoking variable was 50,11 ± 80 P/Y in G1 and 56,47 ± 37P/Y in G2. Average tobacco consumption, Charlson's index, sputum purulence, FEV1, mMRC, mean hospital stay, SGQR, CRP, and exacerbations during the past year were, respectively: 3.75 ± 7.44, 48.7 ± 6 l / s, 3 ± 14, 44, 48%, 48.7 ± 6 l / s, 3 ± 14, 08 days, 34,51 ± 14,23, 25, and 0.89 in G1 compared to 4.26±2.98, 40.2±3 l/s, 4±66, 64%, 12 days, 46,11 ± 74.39, 38, and 1.88 times in G2.

## 3 DISCUSSION

COPD is a serious public health problem and a major cause of chronic morbidity and mortality in the world [10]. A study [11] found that the prevalence of COPD is 4.9% in Algeria. On the other hand, the incidence of bronchiectasis in Algeria is unknown; however, it is estimated that tuberculosis remains the greatest cause of bronchiectasis in the country.

The causes and mechanisms of bronchiectasis in COPD are still not clear.

One hundred twenty patients hospitalized for exacerbation of COPD were included in our study. These cases were divided into 2 groups: group G1 having 91 patients (not having associated bronchiectasis) and G2, including 21 patients with a combination of the two pathologies. The prevalence of this association was 18.75%. In our study, we included not only moderate to severe COPD cases but also mild cases, which could explain this prevalence. This prevalence was similar to the one found in a study conducted by M. Bafadhel [12] and was reported by Yong-hua Gao [13].

The increasing availability and the use of HRCT in COPD indicated that the association of this condition with bronchiectasis is frequent: *i.e*., from 4 to 57% [14-16]. This prevalence varied according to regions and diagnostic criteria. In 71.42% of cases, bronchiectasis was located in the lower lobes, and it was cylindrical type in 80.95% of cases. In 76.19% of cases, it was bilateral. These characteristics were also reported by other studies. The basal seat, the cylindrical type, and the bilateral aspect of bronchiectasis were found, respectively, in 66.6%, 81.8%%, and 72.8% of cases in the study conducted by Eman O [17]. These results were also reported in other studies [18, 19]. The involvement of the lower lobes may reflect gravity-dependent retention of infected secretions [20].

The socio-demographic characteristics of the patients were comparable between the two groups. Our population was predominantly male; we only had 04 women out of the 112 patients included in the study. This gap is explained by less smoking by females, which is not frequent in Algeria and remains a taboo. It explains the gender distribution of COPD in Algeria to be still dominated by males. However, no significant difference was reported in their average age (p = 0.378). G1 patients had a mean age of 70.69 years compared to 68.8 years in G2. Tobacco is the most important risk factor for COPD, causing the two etiopathological components of the disease, obstruction and the decline of respiratory function [21]. It plays an important role in the onset of bronchiectasis by altering the means of defense of the lungs. It promotes chronic colonization in the respiratory tract, which triggers a cascade of events, leading to progressive deterioration of the bronchial wall and the manifestation of bronchiectasis. Smoking was noticed in all patients. In G150.11 PY vs. 56.47PY in G2, there was no significant difference (p=0.249). Tobacco is not a determining factor in the association between COPD and bronchiectasis. This observation was reported in the study conducted by C. Habouria [22] on 100 patients, in which tobacco was reported as 67.5 ± 10.5 PY in G1 and 64.3 ± 10.03 PY in G2, (p=0.128). On the other hand, Eman. O [17] found a significant relationship (p= <0.001) in a study involving 69 patients with moderate to severe COPD. In 2017, GOLD [10] considered bronchiectasis as comorbidity. Both conditions are characterized by inflammation and irreversible obstruction. We found a higher Charlson index in G1, *i.e*., 3.75 ± 7.44 vs. 4.26 ± 2.98 in G2 but without a significant relationship (p = 0.412). Nowiński [23] found that COPD patients with and without bronchiectasis had a similar Charlson index (2.5 vs. 2.1, p=0.05). Another study on comorbidities involved 05 phenotypes (ACOS, chronic bronchitis, COPD with bronchiectasis, emphysema, and frequent exacerbator), Badawy [24] found, in the COPD bronchiectasis group, a 52.4% frequency of cor pulmonale, 42.9% of systemic hypertension, and 38.1% of depression. During the hospitalization of these patients for exacerbation, we noticed that for the Anthonisen index, Type 1 was more frequent in G2, and stroke in 44 patients (48.35%) compared to 13 (61.90%) in G1 (p = 0.521). The significant variables associated with the presence of bronchiectasis found in our study include FEV_1_, mMRC, sputum purulence, length of hospital stay, QSGR, C-reactive protein, and exacerbations in the previous year. These seven variables were included in the logistic regression analysis Table **4**. Multivariate logistic regression analysis showed that FEV_1_ in the COPD group associated with bronchiectasis was more severe (48.7 ± 6 l/s vs. 40.2 ± 3 l/s) (OR=4.3187; 95% C.I **=**2.6301 - 6.8740; p <0.017).

The severity of the respiratory deficit is correlated with the presence of bronchiectasis in COPD. Indeed, many authors reported a linear correlation between the severity of COPD and bronchiectasis [22, 25, 26].

Bronchiectasis with the stagnation of the secretions, which favors the chronic colonization of the airways, also favors sputum purulence, which is correlated with the presence of bronchiectasis in our study (OR=1.4150; 95% C.I **=**0.5012- 2.4189; p <0.029). The sputum purulence has been found to be associated with the presence of bronchiectasis, as reported by several authors [27-29]. Inflammation plays an important role in the etiology of both conditions. One of the essential markers thereof is C-reactive protein (CRP). We found a significant correlation (OR=0.9662; 95% C.I **=**0.2187- 1.8286; p <0.001) between an increased level of CRP and the presence of bronchiectasis (38 in GP 2 *vs*. 25 in GP1). A. Ben Saad [30] conducted a retrospective study on 423 cases of COPD, of which 84 had bronchiectasis, and no CRP difference in the two groups with or without bronchiectasis was reported. Tulek. B [31] and M.Garcia [32] also reported in their respective studies a significant Odds Ratio relationship. Regarding the average length of hospitalization, Qihong Yu [33] reported that the association of bronchiectasis with COPD does not appear to increase the length of hospital stay for an exacerbation of COPD (12.86±5.07 days in G2 *vs*. 11.56±4.06, p=0.113). The severity of the exacerbations, particularly those due to bacterial infections or pseudomonas aeruginosa, plays an important role [34] in predicting a longer treatment and hospital stay. We found that the patients in G2 had a significantly longer hospital stay than G1 patients (12 *vs*. 8 days) with OR= 3.9617; 95% C.I =0.8972- 5.4633 (p=0.014).

On the other hand, A.Molino [35] and G.S.Muñoz [36], in their studies, reported the existence of this significant relationship.

The quality of life is found to be significantly impaired in patients with COPD and associated bronchiectasis. The total score of the Saint Georges questionnaire was higher in G2 group (34.51 ± 14 *vs*. 46.11 ± 74). (OR= 1.9442 95%C.I **=**0.3167- 3.7410;p = 0.032).

D. Carrillo [37] conducted a study in which the COPD Assessment Test (CAT) was performed on 96 patients with bronchiectasis (without COPD). The study of the quality of life and the research on possible depression should lead to proposing a specific treatment in the therapeutic protocol of these comorbidities.

Frequent exacerbations are a hallmark of the association between COPD and bronchiectasis [38-41]. In our study, the number of exacerbations during the past year was higher in the bronchiectasis group.

## CONCLUSION

Association between COPD and bronchiectasis could have important clinical implications since both diseases have different and complementary therapeutic approaches. The diagnosis of bronchiectasis should be more efficient in patients with COPD having a severe respiratory deficit, purulent sputum, accelerated CRP, and deterioration in the quality of life.

Both chronic conditions seem to combine and worsen the psychological impact. The study of the quality of life considering this association is rare, indicating the need for further research.

## Figures and Tables

**Fig. (1):**
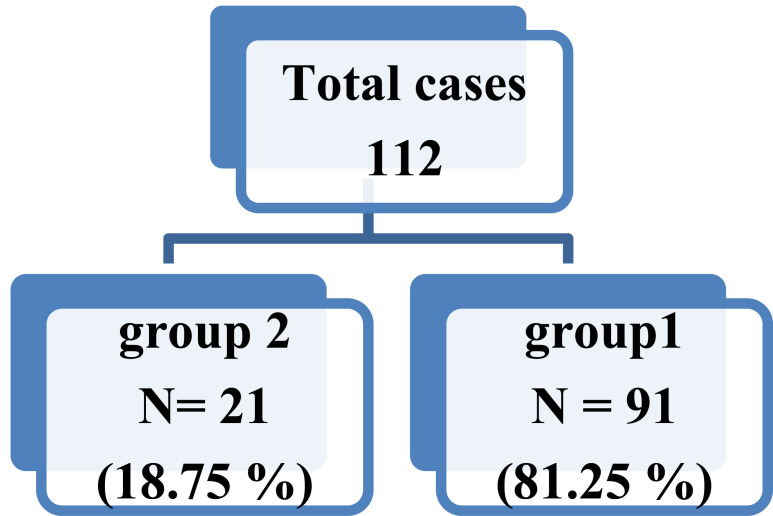
Frequency of bronchiectasis in patients with COPD.

**Table 1:** Characteristics of the study population.

**Setting**	**Features**
Population	112
Age	69.49 Ans ± 8.15 ans
Sex	108 M - 4 F
Smoking	53, 21± 48 P/Y
BMI	20.49 ± 87 m^2^/kg
COPD	Moderate 22.5%
	Severe 43.6%
	Very Severe 33.9%
FEV_1_	42,55 ± 18,90%
BODE	5,54 ± 2,63
6MWT	273,79±112,79 m
I. Charlson	3,99 ± 2,32
mMRC	2.78 ± 0.79

**Table 2:** Characteristics of bronchiectasis on CT in group 2 (COPD associated with bronchiectasis).

**Characteristics**	**N° (%)**
***N_O of Patients_***	21 (18.75)
***Location***	
Upper lobe	06 (28.58)
Lower lobe	15 (71.42)
***Type***	
Cylindrical	17(80.95)
Sacular	03(14.28)
Mixed	01(04.77)
***Bilaterality***	
Bilatéral	16(76.19)
Unilateral	05(23.81)

**Table 3:** Characteristics of bronchiectasis in both groups.

Characteristics	Goup 1	Goup 2	P-value
N_O_	91	21	-
Age	70.69y	68.28y	0.378
Sex			
Male	87	21	-
Female	04	-	
Smoking	50, 11P/Y	56, 47P/Y	0.249
Charlson index	3.75±7.44	4.26±2.98	0.412
FEV_1_	48.7±6 l/s	40.2±3l/s	0.017
mMRC	3±14	4±66	0.031
Purulent sputum	48%	64%	0.029
Anthonisen Index	-	-	0.521
Type 1	13 (10.83%)	44 (48.35%)	-
Type 2	39(42.8%)	04(3.33%)	-
Type 3	08(8.8%)	04(3.33%)	-
Length of hospital stay	08 days	12 days	0.014
SGQR total	34,51 ±14,23	46,11±74.39	0.032
CRP	25	38	0.001
Exacerbations in previous year	0.98	1.48	0.041

**Table 4:** Multivariate logistic regression analysis for statistically significant variables (p<0.05).

	**OR**	**95%C.I.**	
FEV_1_	4.3187	2.6301	6.8740
Purulent sputum	1.4150	0.5012	2.4189
Length of hospital stay	3.9617	0.8972	5.4633
SGQR	1.9442	0.3167	3.7410
CRP	0.9662	0.2187	1.8286
Exacerbations in previous year	0.7523	0.1437	1.5562

## Data Availability

Not applicable.
